# Vulnerability to climate change of islands worldwide and its impact on the tree of life

**DOI:** 10.1038/s41598-019-51107-x

**Published:** 2019-10-09

**Authors:** Simon Veron, Maud Mouchet, Rafaël Govaerts, Thomas Haevermans, Roseli Pellens

**Affiliations:** 1 Institut de Systématique, Evolution, Biodiversité (ISYEB UMR7205), Muséum national d’Histoire naturelle, CNRS, Sorbonne Université, EPHE, Université des Antilles, CP 51, 57 rue Cuvier, 75005 Paris, France; 2 Centre d’Ecologie et des Sciences de la Conservation (CESCO UMR7204) MNHN, CNRS, Sorbonne Université - CP135, 43 rue Buffon, 75005 Paris, France; 3 Royal Botanic Gardens, Kew, Richmond, Surrey, TW9 3AE UK

**Keywords:** Biogeography, Conservation biology, Evolutionary ecology, Macroecology

## Abstract

Island systems are among the most vulnerable to climate change, which is predicted to induce shifts in temperature, rainfall and/or sea levels. Our aim was: (i) to map the relative vulnerability of islands to each of these threats from climate change on a worldwide scale; (ii) to estimate how island vulnerability would impact phylogenetic diversity. We focused on monocotyledons, a major group of flowering plants that includes taxa of important economic value such as palms, grasses, bananas, taro. Islands that were vulnerable to climate change were found at all latitudes, e.g. in Australia, Indonesia, the Caribbean, Pacific countries, the United States, although they were more common near the equator. The loss of highly vulnerable islands would lead to relatively low absolute loss of plant phylogenetic diversity. However, these losses tended to be higher than expected by chance alone even in some highly vulnerable insular systems. This suggests the possible collapse of deep and long branches in vulnerable islands. Measuring the vulnerability of each island is a first step towards a risk analysis to identify where the impacts of climate change are the most likely and what may be their consequences on biodiversity.

## Introduction

It is now widely accepted by the scientific community that the Earth is undergoing drastic climate change due to increased greenhouse gas emissions^1^. Changes in global climate have occurred several times throughout the Earth’s history but have stretched over very long periods of time whereas currently these changes are taking place over the space of a century or less^1–3^. This rapid change, associated with other threats resulting from human activity related to production and consumption^4^, are having a strong impact on biodiversity^5,6^. Considered as one of the five main causes of species and populations losses^7–9^, climate change may lead to direct alterations of natural habitats, forcing species to move from their historical range, adapt to new environmental conditions, find refuge in unaltered microhabitats or may lead to species extinction^3,10–13^. Importantly, climate change acts in synergy with other human-induced threats, such as land use intensification or biological invasion, increasing their effects.

In this context, island biodiversity requires specific attention for several reasons. Insular communities, because they are spatially segregated and have evolved in isolation, are characterized by extremely high rates of endemism^14,15^. Although they occur on less than 5% of the Earth’s terrestrial area, island plants and vertebrates have an endemic richness that may exceed that of mainland species by a factor of 9.5^15^. Island biota are also very prone to extinction: around 80% of past extinctions and a third of threatened terrestrial species are found on islands^16^. Past extinctions were probably triggered by invasive species, the naïveté of insular species, and the high range-restriction of some populations^17–22^. Although climate change might impact island biota in many different ways, it is likely that sea level rise and climate shifts will be of major importance due to their direct association with the availability of suitable habitats for terrestrial organisms. Sea level rise is expected to lead to the submergence of several islands^2,23^. Sea level rise may also increase coastal erosion and saline water intrusion, impacting natural habitats^24,25^. This means that elevation, area, and the complexity of shoreline inlets are of major importance regarding vulnerability to sea level rise^26,27^. As for climate shift, it includes changes in the frequency and intensity of extreme weather events (e.g. droughts, storm surges, hurricanes), as well as altered patterns of seasonal and mid-term weather systems. This leads to the displacement of suitable climatic conditions to different altitudinal or latitudinal ranges, which implies that for some island species, suitable climates may only be found far beyond their limits^28^. Island size and topographic heterogeneity will then play a central role in preserving habitats where species could survive. When suitable habitats disappear, species that are able to adapt or/and have advantageous traits, e.g. flowering times that can track temperature shifts^29^, will probably be naturally selected under the new environmental conditions, whereas others may go extinct. Shifts in climatic conditions may then promote a cascade of local extinctions.

Despite the importance of island biota to global biodiversity, the impact of climate change on insular biodiversity has just begun to be investigated^23,28,30–34^. The few existing studies focused only on species richness, and the lack of knowledge of other biodiversity currencies certainly limits our understanding of this impact. In fact, species richness alone does not provide information regarding certain crucial aspects for species conservation such as evolutionary history^35^. Thus far, we do not know how climate change will impact the tree of life. Will it lead to the loss of deep and/or unique evolutionary branches, or to random pruning? Can we expect a certain tendency towards resilience, as observed on continents^36,37^? If so, for what reasons?

To begin to understand these issues, we designed a study to estimate island relative vulnerability to climate change and assess its impacts on the monocot tree of life. Relative vulnerability allows to identify islands which may be the most threatened by climate change at a world scale. These issues are particularly important for this major group of plants, which has a worldwide distribution and high species richness and endemism on islands. Monocots also provide a wide range of products to mankind. We used the phylogenetic diversity (PD) measure from^38^ to measure the expected PD at risk of being lost^39,40^. PD is more than a simple assessment of the evolutionary aspect of biodiversity as it may highlight current and future benefits provided by biodiversity to people and ecosystems, i.e. option-values^41,42^. Moreover, the loss of PD can be much higher than the loss of species richness (SR) if (i) closely related species go extinct (thus threatening not only terminal branches but also deep branches shared by a set of species), or (ii) if species on long and isolated branches disappear^38,43,40^. Patterns of PD loss may then help identify which parts of the tree of life are the most at risk from climate change. We analysed 1,497 genera distributed across 5,565 islands from across the globe. This is also the first global scale study of this kind to integrate regional variations of climatic change as estimated by the Intergovernmental Panel on Climate Change (IPCC).

## Results

### Frequency and distribution of vulnerability to climate change (step 1)

**Figure 1:**
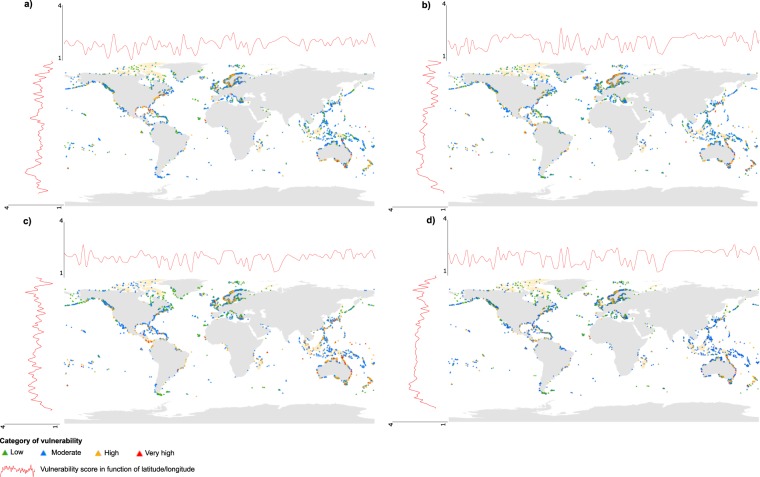
Worldwide distribution of the 5,565 islands with native monocot species and their classification according to four categories of vulnerability (low, moderate, high, very high) to threats related to climate change: (**a**) sea level rise (**b**) shift in temperature, (**c**) shift in rainfall, and (**d**) global vulnerability to climate change. Red lines represent the fitted levels of vulnerability scores (cubic smoothing spline) along longitudes and latitudes.

Over the 5,565 islands studied, the most represented category was “moderate vulnerability”, followed by low, high and very high vulnerability, respectively, for all the types of threats. In general, global vulnerability was correlated to sea level rise, shift in temperature and shift in rainfall, however, scores for these three threats had lower correlations with each other (Table 1). Islands that were most vulnerable to threats from climate change were more frequently situated at low latitudes and/or in the Southern Hemisphere, e.g. in Australia or in the Pacific Ocean, such as Kiribati and French Polynesia islands (Fig. 1). Yet, some northern islands also had high vulnerability, especially regarding shifts in rainfall (Fig. 1), for example in Norway and Sweden. In total, 54, 109, 165 and 34 islands had a very high vulnerability to sea level rise, shift in temperature, shift in rainfall, and global threat from climate change, respectively (Fig. 1; Supplementary Datasets S1 and S2).

**Table 1:** Correlation coefficients between vulnerability scores for each threat across all islands using Pearson’s correlation test.

	Sea Level Rise	Shift in temperature	Shift in rainfall
*Shift in temperature*	0.69		
*Shift in rainfall*	0.67	0.71	
*Global vulnerability*	0.87	0.90	0.89

All results were significant (p-value ≤ 2.2*10^−16^).

### Spatial distribution of ExpPDloss (step 2)

#### Absolute ExpPDloss

**Figure 2:**
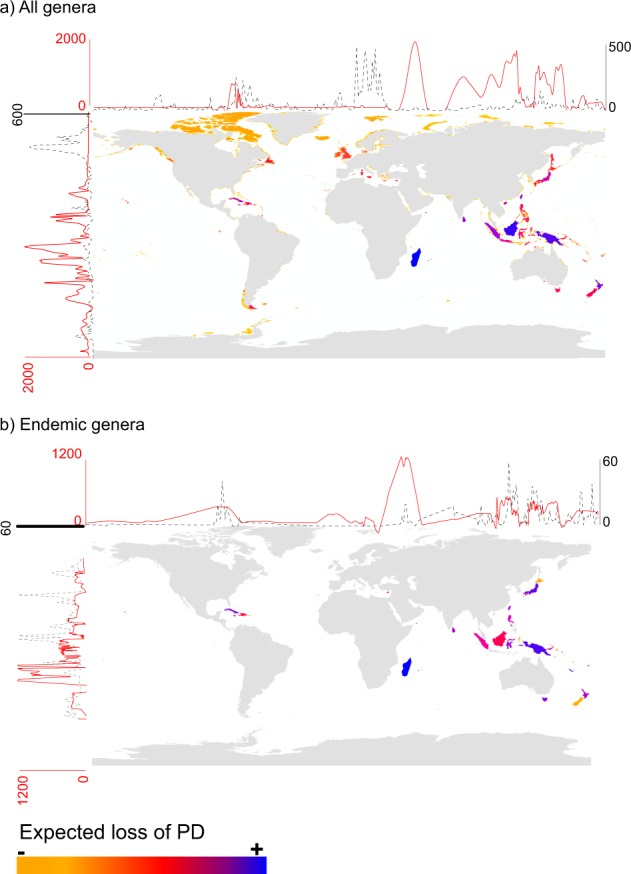
Distribution of the ExpPD*loss* caused by the independent loss of islands when considering (**a**) all monocot genera (n = 1,497) found on islands worldwide (n = 5,565); (**b**) only monocot genera that are endemic (n = 96) to islands (n = 72). Red lines represent the fitted levels of expected diversity loss (cubic smoothing spline) along longitudes and latitudes. Black dashed lines represent the fitted of the number of islands (cubic smoothing spline) along longitudes and latitudes.

Estimates of expected loss of phylogenetic diversity at a global scale (ExpPD*loss*) following local species extinctions indicated that the ExpPD*loss* per island was highest at low latitudes and in the eastern part of the world such as in Madagascar, Borneo, New Guinea, Taiwan, Sri Lanka, Sumatra but with Cuba being one of the most notable exception (Fig. 2; see Supplementary Dataset S1 for the full list of islands and their ExpPD*loss*). The islands whose disappearance would cause the greatest ExpPD*loss* when only endemic genera were included were Madagascar, Sumatra, Tasmania, New-Caledonia, Honshu, Cuba, New Guinea, and Sri Lanka, among others (Fig. 2). When corrected for sampling effort, the relative ExpPD*loss* values changed only slightly when considering either all genera or only endemic genera (Supplementary datasets S1 and S2).

#### Where is ExpPDloss significantly high?

**Figure 3:**
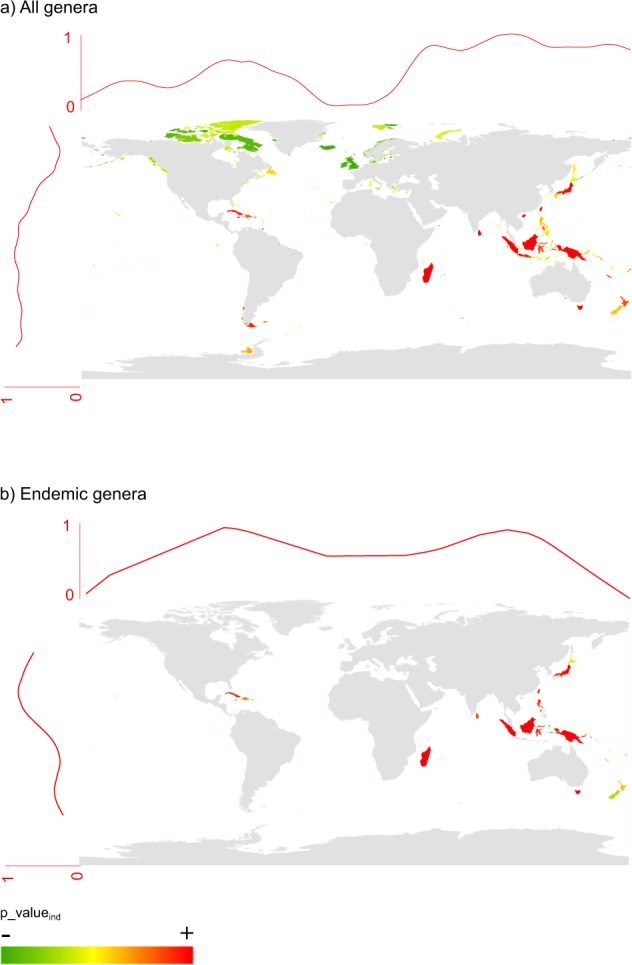
Spatial distribution of islands where ExpPD*loss* is higher than expected at random: (**a**) considering all genera; (**b**) considering only genera that are endemic to islands. Curves at the top and to the left of the maps represent the fitted distribution of p-values_ind_ (cubic smoothing spline) along longitudes and latitudes.

By removing the effect of richness on the ExpPD*loss* using null models (see methods), we found that the most significant losses were predominantly expected at low latitudes and eastern longitudes (Fig. 3). Islands with highly significant ExpPD*loss* were, among others: Madagascar, Borneo, New-Guinea, Tasmania, Sri Lanka, but also Cuba and Tierra del Fuego. For insular endemic genera, significant ExpPD*loss* was found, among others, for Madagascar, Indonesia (Sumatra, Borneo, New Guinea, Sulawesi), the West Indies (Cuba, Jamaica, Hispaniola), Honshu, Sri Lanka, and Tasmania (Fig. 3).

### Would the disappearance of highly vulnerable islands have a large effect on PD? (step 3)

#### Loss of phylogenetic diversity per island and category of vulnerability

**Figure 4:**
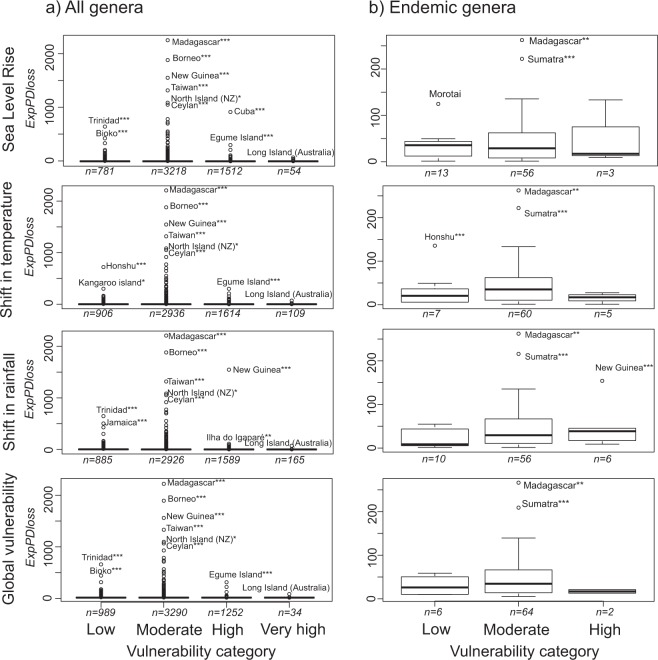
Expected loss of phylogenetic diversity for each vulnerability category and type of threat. *0.95 < p_value_ind_ < 0.99, **0.99 < p_value_ind_ < 0.999, ***0.999 < p_value_ind_ <= 1. “n” is the number of islands per category of threat.

**Figure 5:**
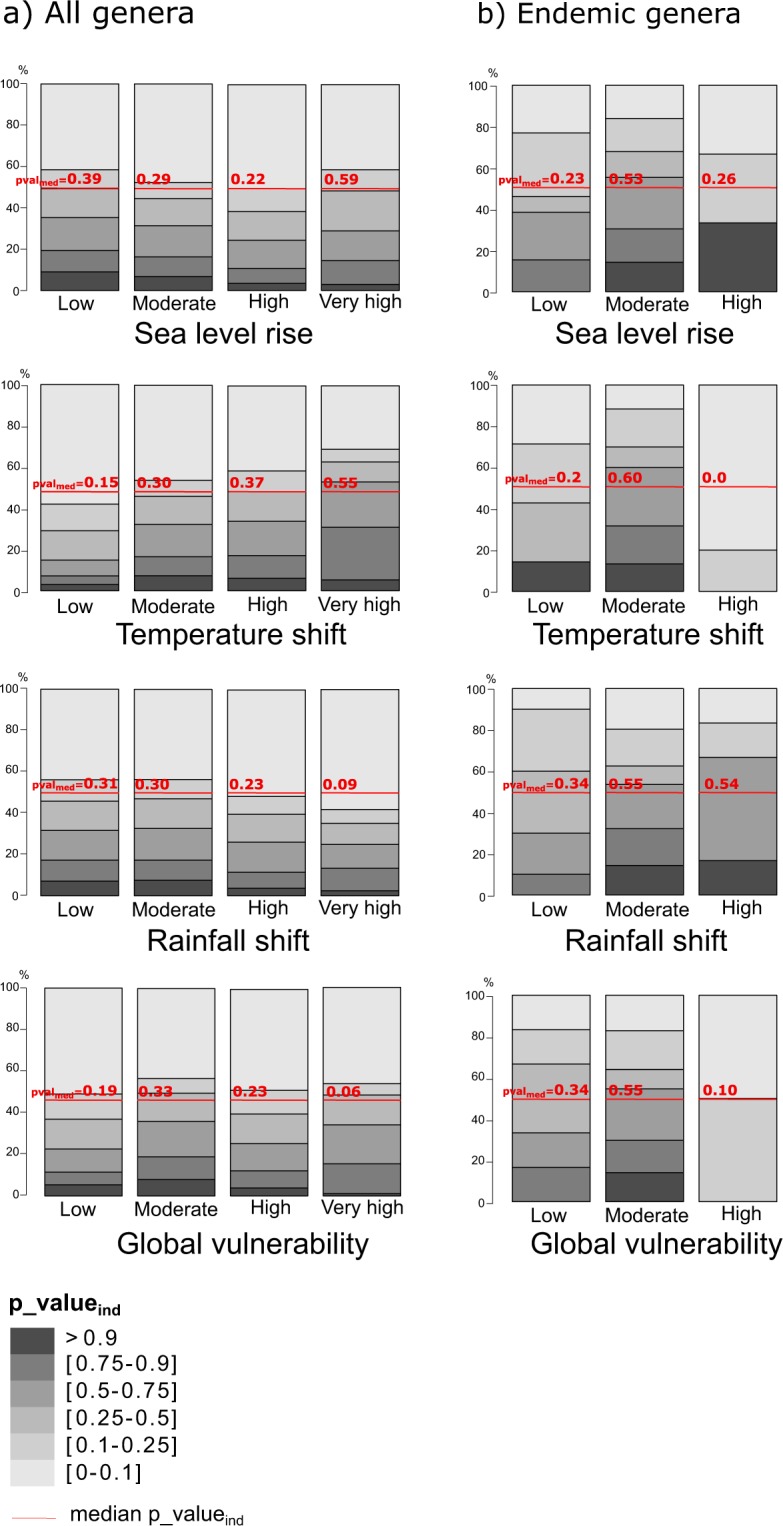
Distribution of p_value_ind_ frequencies for each vulnerability category and type of threat. 50% of p_values_ind_ are higher than the median p_value_ind_ and 50% are lower.

We found that, on average, the ExpPD*loss* for each category of vulnerability was very similar across the four types of threats (Fig. 4). However, the highest values of ExpPD*loss* were found for islands with moderate vulnerability (Fig. 4). Notable exceptions include Cuba and New Guinea, which were highly vulnerable to predicted sea level rise and shifts in rainfall, respectively, and whose disappearance would cause large ExpPD*loss*. Significant ExpPD*loss*, i.e. higher than expected by chance (p_value_ind_), was found for only a small proportion of islands among all categories of vulnerabilities (Fig. 5). For shifts in temperature and sea level rise, the median p-value_ind_ was highest in the “very high vulnerability” category (Fig. 5). When considering only endemic genera, the highest ExpPD*loss* was predicted to occur mainly for islands in the “moderately vulnerable” category (Fig. 4). Moreover, only a small proportion of islands had significant ExpPD*loss* (Fig. 5) and they generally had a low or moderate vulnerability to climate change (e.g. Madagascar, large Indonesian islands).

#### Would the extinction of all genera on vulnerable islands cause higher ExpPDloss than extinctions at random?

**Figure 6:**
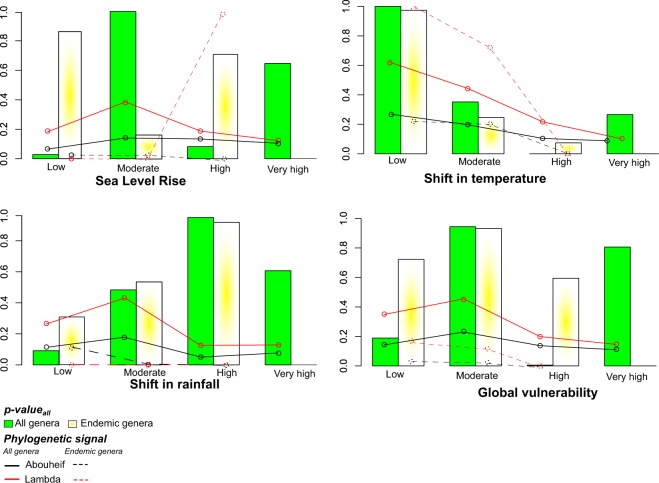
P_value_all_ of the ExpPD_loss_ for each vulnerability category and type of threat. Black and red curves represent the phylogenetic signal of extinction probabilities, calculated using Pagel’s λ and Abouheif’s statistical tests.

We found contrasting results depending on the threat considered. For shifts in temperature, ExpPD*loss* was higher than expected by chance only when all islands with low vulnerability were lost (p_value_all_; Fig. 6). Regarding sea level rise and global vulnerability to climate change, p_value_all_ were relatively high in both islands with moderate and very high vulnerability. As for the effect of a shift in rainfall, ExpPD*loss* was the most significant when highly vulnerable islands were lost. Moreover, for three out of the four threats considered, the loss of genera occurring on the most vulnerable islands would cause higher ExpPD*loss* than expected in 60% to 80% of our randomizations. Yet, we observed that p_values_all_ were poorly congruent with the phylogenetic signal of extinction probabilities (Fig. 6).

Similar results were found when focusing only on endemic genera. ExpPD*loss* was generally greater than by chance with the loss of (i) islands with low vulnerability to shifts in temperature; (ii) islands that are highly vulnerable to shifts in rainfall; (iii) islands with either high or low vulnerability to sea level rise; and (iv) islands with moderate vulnerability to the global threats from climate change  (Fig. 6).

## Discussion

The present change in climate is raising many questions regarding its effect on biodiversity and the conservation measures to be taken to mitigate its impacts^44^. In this study, we focused on islands to investigate the vulnerability to climate change of these segregated spatial units that strongly contribute to the world’s diversity^33^. The main aims were to categorise islands worldwide according to their vulnerability to climate change and to investigate how the estimated vulnerability would impact the tree of life of monocots.

As to be expected, for the set of features considered, the highest vulnerability values were assigned to islands that were small, low, jagged, and located in areas where sea level rise, shifts in temperature and/or rainfall were predicted to be the highest. Yet, the complex interaction between island features and threat levels meant that vulnerable islands were identified across the globe. In particular, low lying islands situated in the Caribbean and the Pacific were highly vulnerable to the effects of sea level rise (e.g. The Cook Islands, French Polynesia, the Marshall Islands)^34,45^. Large predicted shifts in rainfall in North America and Scandinavian countries combined with low elevations and small areas, may explain why many islands that are vulnerable to shifts in rainfall are located in the Northern Hemisphere (although many others are found near the equator and at Southern latitudes).

By estimating the relative threat of climate change on insular biodiversity based on predictions from the IPCC, we identified islands where species may be most at risk in a context of uncertainty^45^. This could then help to anticipate the effects of climate change and establish strategies to preserve biodiversity. Similar analyses could be carried out for other groups of organisms, such as eudicots, mammals^31,34^, or to assess the risks faced by human settlements^46^.

As this is the first study at this scale, some critical features could not be taken into account due to the paucity of standardized information for a large number of islands. We lacked data on the lithology of many islands which is another important factor of vulnerability to sea level rise. For instance, islands with rocky shores are likely to be more stable, whereas islands with sedimentary shores may adjust in shape, size and position over very short periods of time depending on the supply and transportation of sediments^47^. As for reef islands, they might grow following shifts in sea levels (but see^48^). The study of Kench *et al*.^49^ illustrates this complex situation, in that 18 out of 29 coral islands affected by sea level rise had actually grown in size due to new gravel deposits during cyclonic events.

Regarding estimates of vulnerability to shifts in temperature and rainfall, there are at least three main sources of uncertainties and limitations that should be considered. The first is that the scale of global predictions may fail to account for more localised effects and, in some cases, does not allow to distinguish spatially close islands with different environmental conditions^33,50–52^. The second is the uncertainty concerning these estimates for islands located in regions that are largely influenced by major climate drivers with very high variability, and for which even the direction of their impact under climate change is hard to predict (e.g. El Niño Southern Oscillation)^33,53^. The third is the climatic heterogeneity within islands, which is expected to be greater in larger and more elevated islands, with important climatic differences between windward and leeward regions^54^, or along altitudinal belts. This makes that a single vulnerability score may not be sufficient to capture the spatial heterogeneity of threats in large and/or mountainous islands. It must be considered, however, that part of this complexity is comprised in the vulnerability index, as area, altitude and topographic heterogeneity are integrated in the assessment of vulnerability. Our main conclusion regarding this point is that, although the vulnerability scores used here do allow comparing a wide range of islands at a global scale, more research is necessary to understand the effects of climate and topographic heterogeneity on the vulnerability to climate change. As a direction, we suggest focusing on large islands where a significant proportion of the Tree of Life could be lost if some species went extinct (e.g. Madagascar, Borneo, New-Guinea, Cuba, Taiwan, Sri Lanka, Sumatra). In addition, as vulnerability assessments can be conducted at different scales, one prospect would be to define different levels of vulnerability depending on conservation objectives. In this framework, our approach could for instance correspond to “level 1” based on island typology, level “0” could refer to the vulnerability of archipelagos, “level 2” to the vulnerability of habitats etc.

In the Global Assessment Report on Biodiversity and Ecosystem Services released by the Intergovernmental Science-Policy Platform on Biodiversity and Ecosystem Services (IPBES), PD is now recognised as a key indicator of one of “Nature’s Contributions to People” (NCP 18 - maintenance of options)^55^. Indeed, PD provides options for future generations and for organisms to adapt in a changing environment^38,41,42^. The IPBES therefore highlighted that the loss of phylogenetic diversity implies irreversible losses of options to humanity^55^. In this context, assessing the impacts of island vulnerability to climate change represents a first step to provide knowledge for conservation actions aiming at the reduction of irreversible losses.

The first analyses of our research show that extinctions on the most vulnerable islands would remove relatively little insular PD. Indeed, vulnerable islands generally have small size and low elevation which are factors of low species richness^56^, conducting to reduced loss of PD^40^. By contrast, islands that contribute the most to loss of PD were only moderately threatened by climate change. This was the case for Madagascar, Borneo, Honshu, Sri Lanka, Sumatra, New Caledonia, which all harboured high rates of endemic and evolutionary unique genera (Supplementary Method S2)^15^. These islands are large and environmentally diverse which fosters high richness and endemicity but also offers greater opportunities for plants to track their climatic preferences^15,57,58^.

Nonetheless, the link between ExpPD*loss* and taxon richness may hide the fact that some long and/or deep branches of the tree of life may occur on vulnerable islands. By explicitly removing this association using comparisons with null models, we found that some highly vulnerable islands – although not all of them – may lose more PD than expected by chance. This is particularly true for islands that are highly vulnerable to sea level rise and temperature shifts, which is probably due to their greater frequency at low latitudes, where some range-restricted taxa representing relatively large amounts of PD occur (Supplementary method S2). This reinforces the claim that highly vulnerable islands could be home to evolutionary distinct, rare and endangered genera and that climate change would increase their likelihood of extinction^31,34^.

These outcomes correspond to a worst-case and island-centred scenario in which all genera on an island would become extinct, and in which the probability of extinction of a genus is proportional to the number of islands it disappears from. We acknowledge that sensitivity to climate change is highly complex and depend on many factors such as species ecological preferences and tolerances, traits (e.g. phenology), dispersal potential (e.g. among islands of an archipelago) or biotic interactions. Species-centred and trait-based approaches remain therefore essential to understand the responses of species to threats from climate change but may be more relevant at regional or local scales. Finally, we also encourage the mobilization of data hosted in natural history collections and complementary field sampling in islands where impacts of climate change are expected to be high. This would contribute to include several islands that were discarded from the present analysis due to lack of appropriate data, as for example, many islands along the entire coasts of South Asia, North Africa, in the Red Sea, or in the Maldives (only a single island of the archipelago could be included here).

The simulation of the extinction of all genera occurring on vulnerable islands showed one more facet of the consequences of climate change. If genera occurring on vulnerable islands went extinct, the monocot tree of life would be pruned to non-negligible proportions (i.e. causing higher ExpPD*loss* than random). Several hypothesis can be evoked to explain this result. The first is that it is driven by the loss of the most evolutionarily distinct and/or range-restricted genera. Nonetheless, the different tests performed here showed that the expected loss of the most evolutionarily distinct genera and the expected loss of genus richness were rarely significant when all vulnerable islands were lost (Supplementary method S2). A second potential explanation for significant ExpPD*loss* on vulnerable islands would be that genera are closely related^43,40^. However, the phylogenetic signal of extinction risks was relatively low on vulnerable islands, indicating that genera threatened by the loss of the most vulnerable islands tended to be randomly distributed among the tips of the phylogeny. The rejection of these hypotheses leads then to consider two other possibilities. The first is that certain range-restricted genera are clustered in species-poor clades, thus threatening the corresponding deep branches, whereas many others are over-dispersed, i.e. some deep branches also support safe taxa. The second explanation is that some relatively long branches are threatened, although many other evolutionarily distinct genera are secured. These hypotheses that there is a relatively low number of deep and long branches at risk is supported by the moderate frequency of cases where ExpPD*loss* is higher than random. Overall, these findings show that, as also observed by^36,59^, taxa that are vulnerable to climate change may be found either on long or short branches, and whereas some are over-dispersed in the phylogeny, others are clustered in particular clades.

Previous studies considering the impacts of climate change on PD^36,37,59^ were conducted on a continental scale. They showed that climate change could lead to spatial homogenization of PD and that the tree of life would be, to a certain extent, resilient to this threat. In comparison, our study highlights the need to consider the specificity of island systems when devising conservation strategies. Water expanses may constitute a strong barrier against species dispersal. This implies that local extinctions, rather than shifts in the distribution range, are more likely, particularly when considering some features of insular biota such as the loss of dispersal ability, low population sizes, and narrow distribution ranges. Consequently, PD loss instead of PD homogenization is expected and insular PD is certainly more threatened than continental PD.

### Conclusion and prospects

To conclude, assessing the vulnerability of islands to climate change represents a first step towards identifying where its impact on biodiversity is most likely to be seen^27,34,45,60,61^. Importantly, by tackling the crucial challenges to predict and mitigate the effect of climate change on biodiversity, this study contributes to issues of global relevance as defined in the policies of the IPCC^1,2^ and the IPBES^62^. We found that (i) sea level rise, shifts in temperature and shifts in rainfall may not cause high absolute ExpPD*loss* at a global scale (ii) however, ExpPD*loss* on some vulnerable islands tends to be higher than if extinctions were random (iii) taxa found on vulnerable islands likely capture some deep and/or long phylogenetic branches. The expected significant loss of monocot PD on vulnerable islands is then another warning that climate change will affect human well-being^63,64^. The results of this study can be used in two ways. First, they can serve as a guide to identify where to act in order to protect the tree of life. While we focused on monocots, other highly diverse insular groups with many endemic species, such as eudicots, would be worth investigated in order to elucidate to which extent these findings can be generalized. Our analysis of islands worldwide provides a chart indicating where to focus to avoid imminent losses. This shows the need of moving from global to local in order to monitor the status of the local populations subtending these evolutionary branches that are under threat, and study ways to avoid their extinctions. The second perspective concerns the synergistic effects of climate change with other threats impacting biodiversity^65^. Even on islands not considered to be vulnerable to climate change, other factors, especially human-induced habitat losses and species invasions, may represent an immediate threat to their biodiversity^66,67^. Future studies should consider the diversity of threats on island biodiversity, including biotic multipliers, and their interactions.

## Methods

To assess the consequences of climate change on island phylogenetic diversity, we proceeded as follows:

1. We calculated the vulnerability of each island to three threats related to climate change depending on some island features
2. We estimated how much phylogenetic diversity (PD) would be lost on a global scale when an island disappears. As the extent of the loss of PD depends on an island’s genus richness, we also estimated whether these losses were significant or not, i.e. whether they were higher than by chance alone.
3. We linked island vulnerability to loss of PD. In particular, we assessed how much PD would be lost when all islands in the same vulnerability category were removed and whether these losses were significant.

The three steps were repeated for all monocot genera occurring on islands and then only for those genera that are restricted to islands, i.e., not observed on the mainland.

### Data

#### Islands

Following previous studies^15,56^, we defined an island as an area surrounded by water whose size is smaller than Australia and excluding islands within continents. Our island database was extracted from the Global Island Database released by the United Nations Environment Program which has information on 180,495 islands from all over the world^62^. We worked at the island but not archipelago scale.

#### Phylogeny and plant occurrences

We used a monocot phylogeny that is well resolved at the genus level for the vast majority of genera^68^. Working at the genus level allowed us to take into account taxa united by solid shared evolutionary history, materialized by shared traits which made them successful on islands and which are therefore the object of frequent evolutionary and ecological research^69,70^. Genera names from this phylogeny were matched with the accepted names found in the eMonocot database and used as a reference to check for the synonymy of plant occurrence names. eMonocot is built by an international consortium of botanists and aims to provide the most reliable information on monocot taxonomy and occurrence.

Plant occurrence points were downloaded from the Global Biodiversity Information Facility portal (GBIF, GBIF.org (09 February 2017) GBIF Occurrence Download https://doi.org/10.15468/dl.kyylbs). We selected all “basisOfRecord” occurences comprised within the island polygon boundaries, defined in the Global Island Database (GID)^62^. We used the taxon name structure from eMonocot to account for possible synonymies in the GBIF dataset and attributed an accepted name following the synonymy list in eMonocot for each record. We excluded all marine taxa.

We then filtered out occurrences of species that are not native to island polygons using the eMonocot database which provides information on a species’ “native” or “non-native” status. To do so, we attributed a Taxonomic Databases Working Group polygon (TDWG; scale used in the eMonocot database^71^) to each island polygon used in our analysis^62^. All GBIF occurrences associated with a TDWG polygon and corresponding to a non-native species in the eMonocot database were excluded, which could sometimes lead to the exclusion of a genus.

However, with this method we could not identify the status of taxa with occurrences where information regarding the “native” or “non-native” status was missing in the eMonocot database. A second test was carried out for those species. For each, we delimited a polygon defined by the maximal and minimal latitudes and longitudes of their known native range. We then excluded the occurrences of species found outside their respective polygon.

Crossing data from large databases - the GBIF, eMonocot, and GID^62^ databases – we compiled 2,550,396 occurrences representing 15,901 species from 1,497 genera on 5,565 islands. Although this seems to be a small proportion of the initial sample (180,495 islands), suggesting that more sampling efforts are needed, this represents one of the largest dataset used for large scale conservation studies in islands. The use of eMonocot allowed reducing taxonomic and geo-localization errors and uncertainties, and therefore gave us more confidence in the validity of our data compared to using GBIF alone^72^. It also allowed to exclude non-native species, complete and correct a species’ area of distribution, and check for synonymies.

## Step 1 - Assessing Island Vulnerability

First, we assessed the vulnerability to climate change of the 5,565 islands harbouring native monocot species, by evaluating how they will be impacted by sea level rise, shifts in temperature, and shifts in rainfall. Vulnerability is a concept used to evaluate the potential impacts of climate change on a system^61,60^. The factors of vulnerability considered and the calculation of vulnerability scores are described below.

### Factors of vulnerability

**Figure 7:**
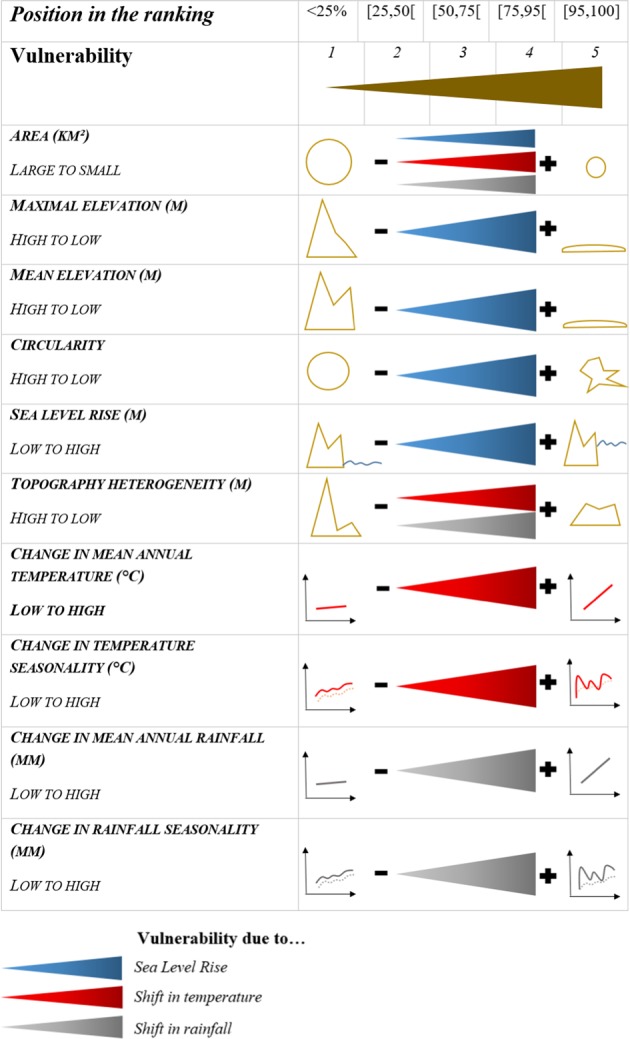
Vulnerability scores for each factor of vulnerability and their associated threat. For each factor, islands were ranked according to the expected influence of that factor on their vulnerability. Islands were then assigned to five classes (from low (1) to high (5)) depending on their ranking position. For example, the highest score was attributed to islands in the top 5%.

To estimate island vulnerability, we used a set of features that are expected to have a strong influence on the effects of climate change on insular biodiversity (Fig. 7)^60,26,34^. Vulnerability to the three threats mentioned above was estimated by considering the following factors:

- *Sea Level Rise*. We estimated the vulnerability to this threat using the values of sea level rise predicted by the IPCC^1^ and considering four island features: area, maximal elevation, mean elevation and circularity. The choice of these variables was based on the reasoning that a large area, high elevation, and roundness (i.e. smallest possible perimeter) increase the proportion of the island surface not affected by floods, erosion, and saltwater intrusion. This consequently reduces the possibility of sea level rise affecting the island’s entire biota^26,27^.
- *Shift in temperature*. We assessed the vulnerability to shifts in temperature by calculating the difference between predicted and current mean annual temperature and between predicted and current temperature seasonality (defined as the standard deviation of mean monthly temperatures). We also considered that the vulnerability of an island to a shift in temperature will depend on area and topographic heterogeneity. Indeed, a large and topographically heterogeneous island may comprise a wider range of climates and thus is more likely to maintain suitable climates in at least part of its territory.
- *Shift in rainfall*. We assessed the vulnerability to shifts in rainfall by calculating the difference between predicted and current values of mean annual rainfall and the difference between predicted and current rainfall seasonality. We also considered factors related to area and topography, which are related to the likelihood of maintaining suitable climates (as with the vulnerability to shifts in temperature).

Current climatic variables were extracted from WorldClim version 2.0^50^. Predicted temperature, rainfall and sea level rise values came from the IPCC^1^ (provided by WorldClim version 1.4) and represent climate projections from global climate models based on four concentration pathways of representative greenhouse gases. In order to determine which scenario(s) to use, we performed a series of preliminary tests to assess the impact of each scenario on estimates of island vulnerability. Results indicated that islands were ranked similarly in their vulnerability to climate change under different scenarios. Although scenarios differed greatly in their estimates of sea level rise, temperature, and rainfall changes, they did not differ much regarding the spatial distribution of these changes (i.e. regions expected to experience the most change were roughly the same). We therefore selected the scenario that considers 2.6 Watts per square meter of cumulative emissions of human greenhouse gases. This corresponds to the most optimistic scenario, where temperature rise remains below 2 °C above pre-industrial values^1^, and is the proposed target of the Paris Agreement on Climate Change.

Elevation and area data were extracted from Weigelt *et al*.^73^ and GID^62^ databases, respectively. Island circularity was estimated as the ratio between the squared perimeter and the area divided by 4π, with values ranging from 0 to 1 (a perfect circle having a value of 1).

### Calculation of island vulnerability

We estimated the vulnerability of each island to each threat, i.e. sea level rise, shift in temperature and shift in rainfall. For each factor associated with a particular threat related to climate change, islands were ranked according to the expected influence of that factor on island vulnerability (Fig. 7). We then attributed a score between 1 (low rank) to 5 (high rank) to each island depending on its position in the ranking. For each threat, each island was given 4 or 5 scores (for shifts in temperature/rainfall and sea level rise respectively) (see above; Fig. 7). The vulnerability of an island to a threat is quantified as the mean of these scores.

## Step 2 - Loss of Phylogenetic Diversity When Islands are Lost Independently

As a second step, we measured the amount and significance of global PD loss following the independent loss of islands, i.e. local extinction of all taxa on a given island. The aim was to identify which islands would have the greatest impact on the tree of life if they were lost.

### Measuring expected PD loss per island

First, we estimated how much PD would be lost across the entire phylogeny of monocots if all genera occurring on a given island went extinct. To do so, we simulated the “loss” of an island, i.e. local extinction of all its taxa. We then estimated the probability of extinction for each genus on an island as the expected proportion of the genus’ global range lost if the island would disappear. Geographic range has been found to be one of the main predictors of species extinction risk, but presence-only data and the huge number of islands and species studied here did not allow assessing individual species range size. Yet, changes in the number of localities where a species occurs is also an essential factor of extinction risk and is used in the criteria B of the IUCN Red List threat status. In our analysis, species range was therefore measured as the number of islands a genus occurs on. This posits that each island equally contributes to a genus’ survival and, therefore probabilities of extinction are given by the proportion of native islands from which a genus disappears. Following Faith^39^, each branch of the phylogenetic tree was weighted by the product of the extinction probabilities of all its descendants, *i*.*e*. equal to the proportion of the range lost for each genera found on the “lost” island and equal to 0 for all other genera. The branches supporting genera which had not lost any proportion of their range were considered safe (probability of extinction equal to 0). We calculated the expected loss of insular PD following the loss of a given island as the sum of weighted branch lengths (^39^; Eq. 1). This measure estimates how much PD is expected to be lost on a global scale following local extinctions on a given island. It also allows us to consider the phylogenetic complementarity of extinction risks, i.e. the shared responsibility of taxa to safeguard deep branches^39^.

1 $$ExpPDloss(tree,\,proba)=\sum _{b}{L}_{b}\,\prod _{{k}_{b}}{p}_{{k}_{b}}$$

where *k*_*b*_ designates the *k*^th^ descendant of branch *b* on the tree, *p*_*kb*_ is the extinction probability of the *k*^th^ descendant of branch *b*, and L_*b*_ is the length of branch *b*^39^.

We ran this analysis for every native insular plant genera. Using this measure, the probability of extinction of a taxon was related to the number of islands it was found on independently of its potential occurrence on continents. Here, ExpPD*loss* has to be interpreted as the impact of island disappearance on monocot PD across islands but not on monocot PD worldwide (representatives of these genera may survive on continents). To complement this measure, we estimated ExpPD*loss* from a pruned tree comprising only genera that are endemic to islands, i.e. those found on a single or in several islands but not on the mainland (96 genera from 72 islands). This represents the PD at risk of irreversible loss, because these genera will not survive anywhere else if the islands where they live are lost. Finally, to correct for low sampling on some islands, we corrected ExpPD*loss* using the predicted genus richness for each island as described in Supplementary method S1.

### Measuring the significance of ExpPDloss per island

As absolute ExpPD*loss* may be correlated with genus richness on an island, we tested whether loss of plant PD on each island was higher than expected by chance. To do so, we randomized genera between islands but kept the genus richness of each island and the range of each genus (i.e. the number of islands a genus is present on). For each island and randomization, we calculated the new ExpPD*loss* when that island is lost. Simulations were run 1000 times. For each island, we then calculated the frequency at which the observed ExpPD*loss* was higher than the 1000 ExpPD*loss* values calculated from simulations (p-values_ind_; unilateral test). This procedure allowed removing the dependency of ExpPD*loss* on genus richness while retaining the structure of the data. Expected PD losses from the original data were considered highly, moderately or slightly significant when they fell into the highest 0.1%, 1% and 5% of the distribution values obtained from simulations, respectively.

To identify areas where range-restricted and/or evolutionarily distinct genera co-occur, we also estimated for each island the expected loss of species richness (ExpSR*loss*) and the expected loss of species richness in the most evolutionarily distinct species (ExpSR*loss*_ED_^74^). We calculated absolute values and their significance. Results were often similar to those of ExpPD*loss* and are described in Supplementary method S2.

## Step 3 - Linking Expected PD Loss to the Vulnerability of Islands

### Absolute value and significance of ExpPDloss per island in relation to island vulnerability

The third step of the analysis was to link island vulnerability (step 1) to the expected loss of PD (step 2). We first estimated the *absolute* ExpPD*loss* per island per category of vulnerability. Due to the high number of vulnerability scores we defined 4 categories of vulnerability: low (1 ≤ vulnerability score < 2), moderate (2 ≤ vulnerability score < 3), high (3 ≤ vulnerability score < 4) and very high vulnerability (4 ≤ vulnerability score ≤ 5). From this we identified the islands and the categories of vulnerability where ExpPD*loss* would be the highest.

Second, we linked the *significance* of ExpPD*loss* when islands were lost independently to the category of vulnerability they belonged to. We calculated the distribution of p-values_ind_ (i.e. the departure from the null model described above) and the median p-value_ind_ per vulnerability category. We identified in this way the categories where significant ExpPD*loss* was most frequent.

### Expected loss of PD when all islands with a similar vulnerability to climate change are lost

As a second approach to link island vulnerability to loss of PD, we tested whether loss of *all* islands assigned to a given vulnerability category would lead to higher ExpPD*loss* than at random (p_values_all_). This differs from the previous analyses which considered the *independent* loss of islands (p_values_ind_).

 Here we calculated extinction probabilities for each genus as the proportion of their range that would be lost if *all* islands with a similar vulnerability to climate change disappeared (in step 1 the extinction probability was measured as the proportion of a genus range lost when *a single* island disappeared). To explore this question, we defined the “range” of a genus as the number of vulnerability categories of the islands on which it occurs. For example, if a genus occurs *only* on highly vulnerable islands, its extinction probability would be 1 if all these islands were lost. The extinction probability of a genus occurring on islands from three categories (e.g. very high, high, and moderate vulnerability) would be 1/3 under a similar scenario. We then calculated the phylogenetic signal of extinction probabilities for each category of vulnerability and each type of threat using Pagel’s λ^75^ and Abouheif’s^76^ statistical tests. These tests assume to reflect phylogenetic signal in a Brownian motion model of evolution and are robust when polytomies are present, which is the case here^77,78^.

We then compared ExpPD*loss* when all genera found on islands belonging to a given category of vulnerability were lost to ExpPD*loss* when genera were distributed randomly among categories of vulnerability. In this randomization procedure, the number of genera per category of vulnerability and the number of categories of vulnerability a genus belonged to were retained. This procedure was run 1000 times and we calculated whether the frequency of the observed ExpPD*loss* per vulnerability category was higher than by chance (p_values_all_). This approach was similar to the one used in step 2 but here genera were randomized between categories of vulnerability instead of between islands as distinct units. To account for evolutionary distinctiveness and range-restriction, we also performed a similar analysis for ExpSR*loss* and ExpSR*loss*_ED_ (Supplementary method S2). Moreover, all analyses described above were conducted on all genera occurring on islands and then only on genera restricted to islands.

## Supplementary information

Supplementary information

Supplementary Dataset 1

Supplementary Dataset 1

## Data Availability

Data will be made available following acceptance
