# Supplementary material for: Vulnerability to climate change of islands worldwide and its impact on the tree of life
Source: Sci Rep. 2019 Oct 9;9:14471. doi: 10.1038/s41598-019-51107-x (PMC6785531; doi:10.1038/s41598-019-51107-x)

Vulnerability to climate change of islands worldwide and its impact on the tree of life

Simon Veron[1,2*], Maud Mouchet[2], Rafaël Govaerts[3], Thomas Haevermans[1], and Roseli Pellens[1]

[1] *Institut de Systématique, Evolution, Biodiversité (ISYEB), Muséum national d'Histoire naturelle, CNRS, Sorbonne Université, EPHE, Université des Antilles, CP 51, 57 rue Cuvier, 75005 Paris, France*
[2]*Centre d'Ecologie et des Sciences de la Conservation (CESCO UMR7204) MNHN, CNRS, Sorbonne Université - CP135, 43 rue Buffon, 75005, Paris, France*
[3]*Royal Botanic Gardens, Kew, Richmond, Surrey TW9 3AE, UK.*

[*]Author for correspondence (E-mail: sveron@edu.mnhn.fr; Tel.: +33 1 40 79 57 63; Fax: +33 1 40 79 38 35).

* TH and RP are senior co-authors of this article

Supplementary Method S1: *Correction of expected diversity loss values*

Summary

a. General method to correct expected diversity loss values
b. Variables tested
c. Measuring sampling effort
d. Contribution of variables to species richness
e. References in Supplementary Method S1

a) General methodology to correct expected diversity loss values

GBIF data are biased by the uneven geographic coverage among islands We included this source of uncertainties using a two-step procedure. First, we calculated an index of geographic coverage for each island. To do so, we estimated a prediction of genus richness in each island from a Boosted Regression Trees model (hereafter "BRT"). In this model, the response variable is the number of genera in an island - calculated from the GBIF - and the explanatory variables are a combination of bioclimatic, geographic and historic variables (see Supplementary Method S1(b)). BRT is an ensemble method for fitting statistical models that combines algorithms of regression trees and boosting [1]. We chose the optimum number of trees based on learning rate, tree complexity and bag fraction that we then used to predict values of species richness. We finally calculated an index of geographic coverage for each island as the observed value of species richness over the predicted valued.

In a second step, we performed linear models of ExpPDloss (and ExpSRloss and $ExpSRloss_{ED}$ [Supplementary Method S2]) in function of our index of coverage. Corrected values of ExpPDloss, (and ExpSRloss and $ExpSRloss_{ED}$) were defined as the residuals of this linear model. We performed calculations both to correct losses estimated from all genera found in islands and for genera strictly endemic to islands (no occurrence on continents).

b) Variables tested

To predict values of species richness we tested the effect of geographic, bioclimatic and historical factors:

i. Geographical and physical factors of islands: area (km^2), elevation (meters), minimum distance to continent (km), the proportion of surrounding landmass, latitude and longitude. Species diversity may theoretically increase with area and proximity to continent and these factors were both shown to be among the strongest predictor of plant species richness [2] . As for the proportion of surrounding landmass (SLMP), which accounts for the size and coastline shape of surrounding landmass, it has been found to be a valuable isolation metric to explain island plant diversity at a global scale [3]. Maximal elevation is related to topography and environmental heterogeneity, for example due to temperature decrease with altitude [4]. This was shown to explain a great proportion of insular plant species richness [2]. As for latitude and longitude, they may highlight differences related to the geographic position of islands and especially the diversity latitudinal gradients [5].

ii. Bioclimatic factors: we selected the number of ecoregions occurring in an island, mean annual temperature (C°), mean annual rainfall (mm), temperature seasonality (C°), rainfall seasonality (mm), mean and standard deviation in annual solar radiation (kJ m^{-2} day^{-1}), mean and standard deviation in annual wind speed (m s^{-1}), mean and standard deviation in annual water vapor pressure (kPa), isotherm (C°). Bioclimatic factors may filter out lineages weakly adapted to the environmental conditions of an area [6]. Especially, temperature and rainfall are among the main predictors of plant species richness [2,7,8]. Wind speed may act on plant diversity by favoring long distance dispersal[9], influence plant growth and may select for lineages adapted to cool or harsh wind conditions. Evapotranspiration, measured

from water vapor pressure, and solar radiation are key components related to energy availability. They were both shown to be predictors of plant richness in islands as well as in continents [7], [9]. As for the number of ecoregions it reflects areas with distinct environmental conditions and distinct assemblage of natural communities sharing a large majority of species [10]. Temperature, rainfall, wind speed, solar radiation and vapor pressure data were extracted from Worlclim version 2 [11] and missing data were completed thanks to the GID database [12]. We used the ecoregions defined by the WWF [10].

iii. Historical factors. We used the velocity of past climate change.
The effect of past climate change velocity may depend on the taxon considered but low velocity were generally associated to high rates of endemism at regional scales [13]. Data of velocity of past climate change came from Sandel et al. [13].

iv. Sampling effort: we tested for the effect of the ICE*r* metric described below in Supplementary Method S1 (c).

To avoid collinearity in the models, the following variables, showing high correlations (Pearson correlation coefficients > 0.5), were removed from models: temperature seasonality, isotherm, mean annual solar radiation, standard deviation in solar radiation, mean annual vapor pressure and standard deviation in wind speed.

c) Measuring sampling effort

To estimate sampling effort, we chose the Incidence-based Coverage Estimator (ICE ; [14]) which is estimated from the number of rare species in sub-samples and species accumulation curves. Compared to other estimators of species richness, the ICE may best satisfy the requirements for an ideal species-richness estimator such as the capacity to

reach a stable value independently of sample size, the low sensitivity to sampling patchiness and density. To calculate ICE, we defined a sub-sample in each island as a set of observations obtained at a given date. We then estimated ICE thanks to the R function spp.est found in package ‘Fossil’ [15]. Finally, we calculated the ratio of the observed number of species in an area on the expected number of species estimated from ICE, an index we called ICE_r.

d) Contribution of variables to species richness

To reach convergence, we parameterized BRT with a bag fraction of 0.5, a learning rate of 0.005 and a tree complexity of 8,700 trees. The contribution of each variable is represented in Figure S1

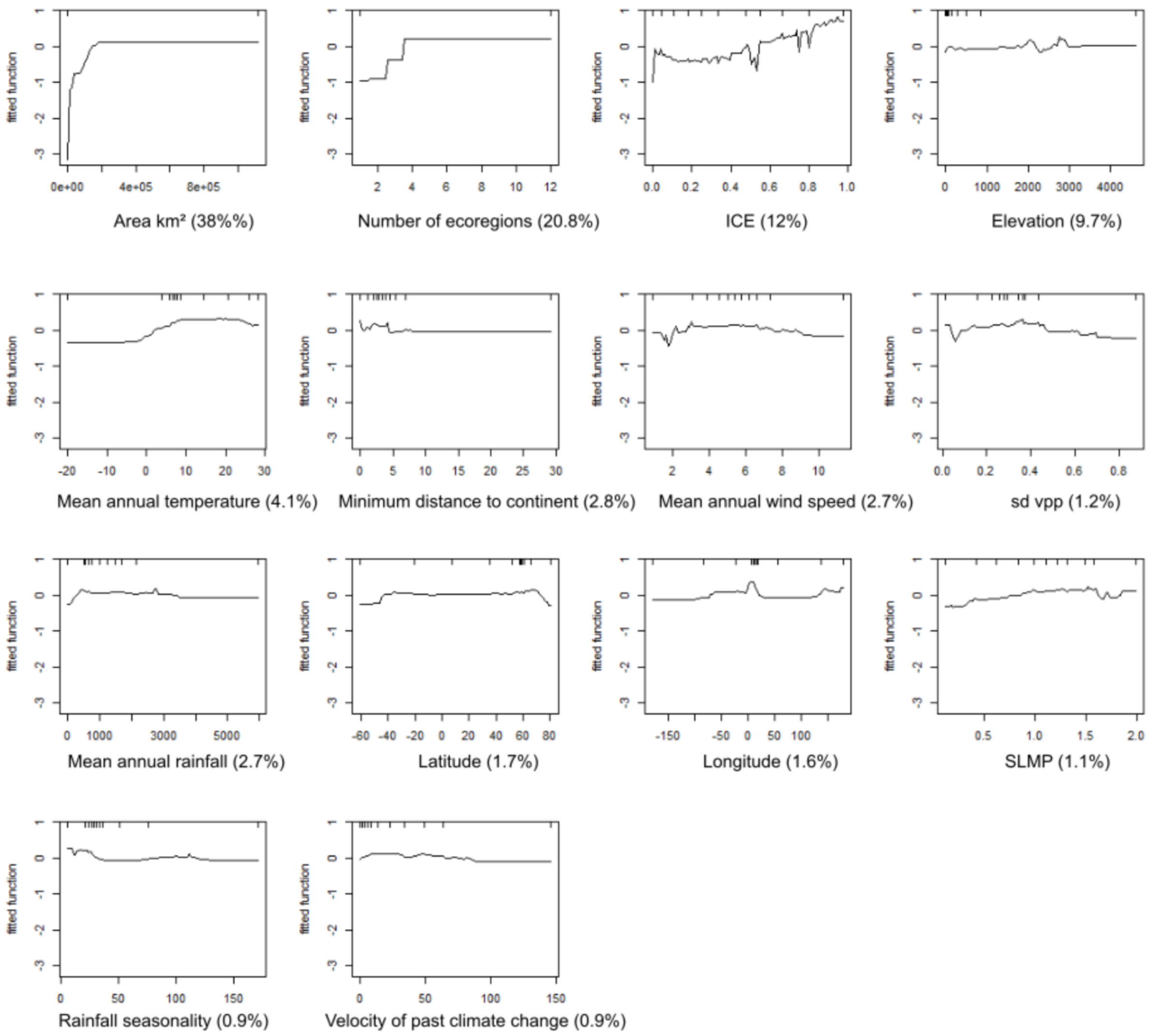

Figure S1: Contributions of biotic variables to genus richness in world islands

e) References in Supplementary Method S1

1. Elith, J., Leathwick, J. R. & Hastie, T. A working guide to boosted regression trees. *J. Anim. Ecol.* **77**, 802–813 (2008).
2. Kreft, H., Jetz, W., Mutke, J., Kier, G. & Barthlott, W. Global diversity of island floras from a macroecological perspective. *Ecol. Lett.* (2007).
3. Weigelt, P. & Kreft, H. Quantifying island isolation - insights from global patterns of insular plant species richness. *Ecography* **36**, 417–429 (2013).

4. Allouche, O., Kalyuzhny, M., Moreno-Rueda, G., Pizarro, M. & Kadmon, R. Area-heterogeneity tradeoff and the diversity of ecological communities. *Proc. Natl. Acad. Sci.* **109**, 17495–17500 (2012).
5. Pianka, E. R. Latitudinal Gradients in Species Diversity: A Review of Concepts. *Am. Nat.* **100**, 33–46 (1966).
6. Webb, C. O., Ackerly, D. D., McPeek, M. A. & Donoghue, M. J. Phylogenies and Community Ecology. *Annu. Rev. Ecol. Syst.* **33**, 475–505 (2002).
7. Currie, D. J. Energy and Large-Scale Patterns of Animal- and Plant-Species Richness. *Am. Nat.* **137**, 27–49 (1991).
8. Kreft, H. & Jetz, W. Global patterns and determinants of vascular plant diversity. *Proc. Natl. Acad. Sci.* **104**, 5925–5930 (2007).
9. Wright, D. H. Species-Energy Theory: An Extension of Species-Area Theory. *Oikos* **41**, 496 (1983).
10. Olson, D. M. *et al.* Terrestrial Ecoregions of the World: A New Map of Life on Earth. *BioScience* **51**, 933 (2001).
11. Fick, S. E. & Hijmans, R. J. WorldClim 2: new 1-km spatial resolution climate surfaces for global land areas: New Climate Surfaces For Global Land Areas. *Int. J. Climatol.* **37**, 4302–4315 (2017).
12. UNEP-WCM. Global islands database. (2013).
13. Sandel, B. *et al.* The Influence of Late Quaternary Climate-Change Velocity on Species Endemism. *Science* **334**, 660–664 (2011).
14. Lee, S.-M. & Chao, A. Estimating Population Size Via Sample Coverage for Closed Capture-Recapture Models. *Biometrics* **50**, 88 (1994).
15. Vavrek, M. Fossil: palaeoecological and palaeogeographical analysis tools. *Paleontol. Electron.* (2011).

Supplementary Method S2: Expected loss of species richness and evolutionary distinctiveness

We calculated the expected loss of SR and its significance as the sum of plant extinction probabilities following the loss of an island.

$$ExpSRloss(proba) = \sum_i p_i$$

where *i* designates the ith species (monocot genera in our study) and p_i denotes its extinction probability.

We assessed the loss of species richness in the most evolutionary distinct species (SR_{ED}) as ExpSRloss calculated in the top 10% of the most evolutionary distinct species. Evolutionary distinctiveness was estimated using the fair proportion index (Redding & Mooers, 2006).

Figure S1. Insular diversity loss caused by the independent loss of islands a) ExpSRloss b) $ExpSRloss_{ED}$ for all monocot genera; c) ExpSRloss d) $ExpSRloss_{ED}$ for monocot genera that are endemic to islands. Graphs above and to the left of the maps represent a fit (cubic smoothing spline) of expected diversity loss along longitudes and latitudes, respectively.

Figure S2. Observed SR loss that is significantly greater than that estimated from the null model (p-values) a) ExpSRloss b) $ExpSRloss_{ED}$ for all monocot genera; c) ExpSRloss d) $ExpSRloss_{ED}$ for monocot genera that are endemic to islands. Graphs above and to the left of the maps represent a fit (cubic smoothing spline) of p-values along longitudes and latitudes, respectively.

Figure S3. ExpSRloss and $ExpSRloss_{ED}$ for each vulnerability category and type of threat. "*": $p\_value_{ind}<0.05$, "**": $p\_value_{ind}<0.01$, "***": $p\_value_{ind}<0.001$.

Figure S4. Observed ExpSRloss and $ExpSRloss_{ED}$ that are significantly greater than those estimated from null models (p-value) for each vulnerability category and type of threat.

Figure S5. P_value$_{all}$ for a) ExpSR*loss* and b) ExpSR*loss*$_{ED}$ for each vulnerability category and type of threat.

Figure S1

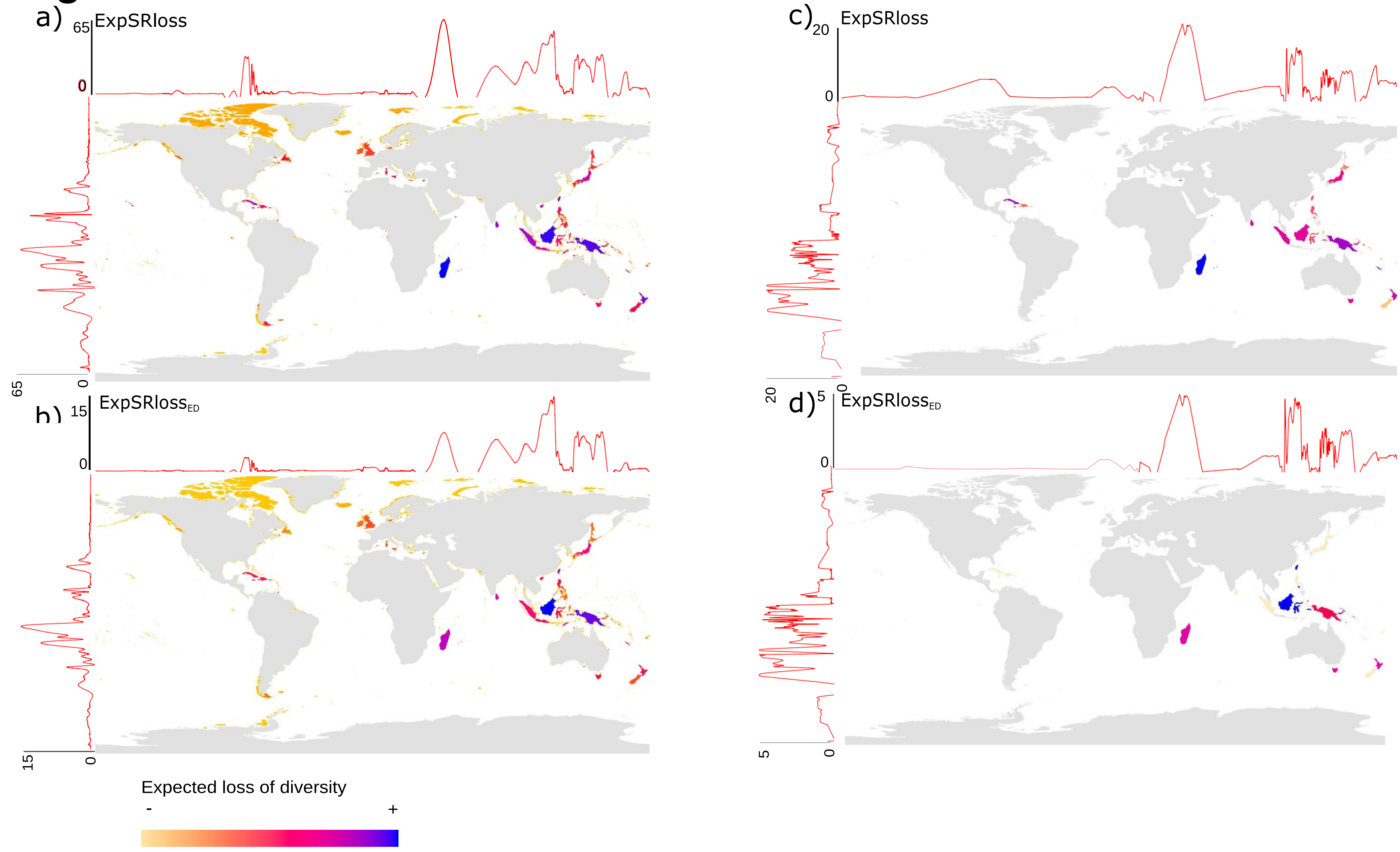

Figure S2

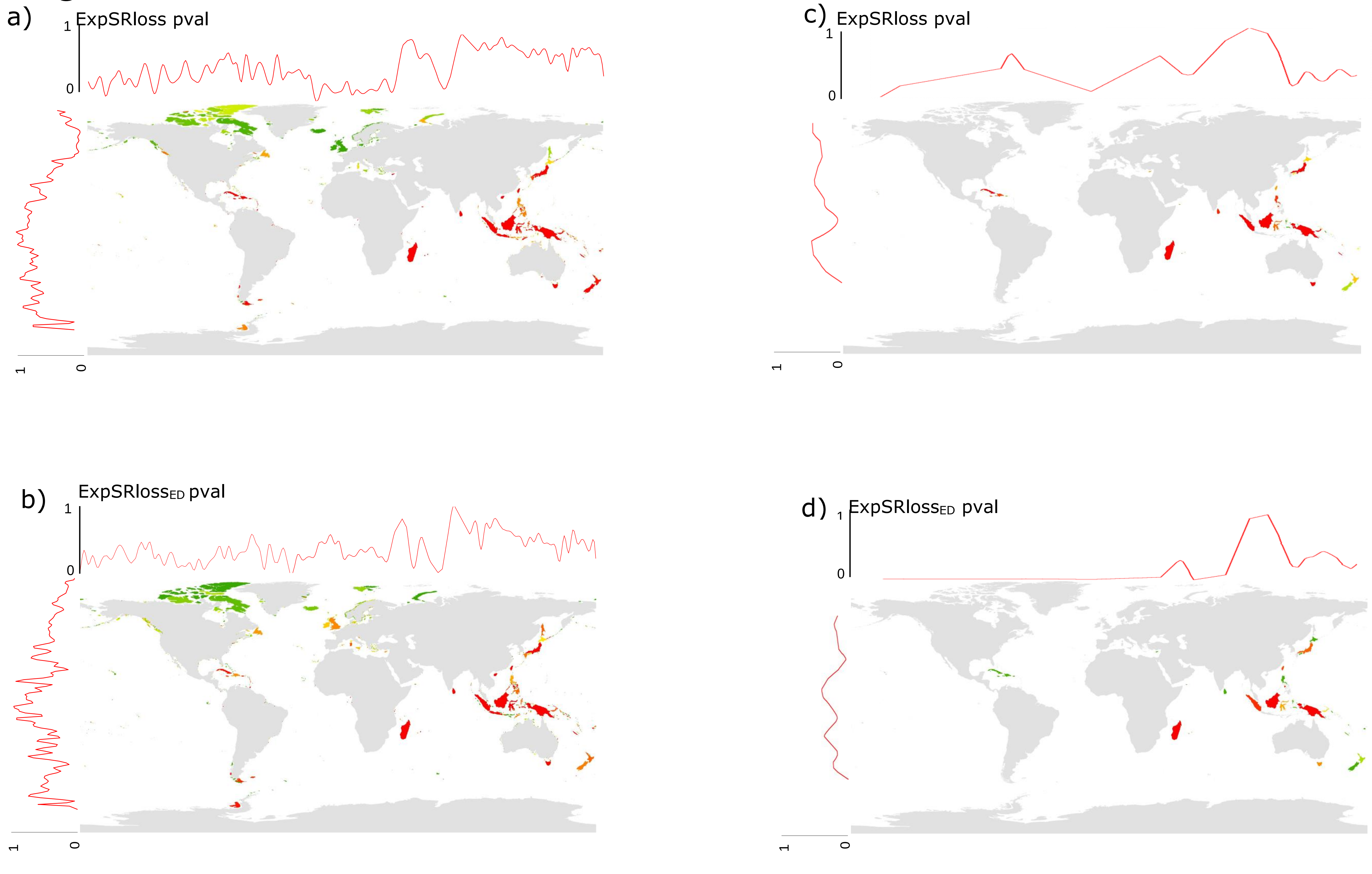

1. Expected loss of species richness

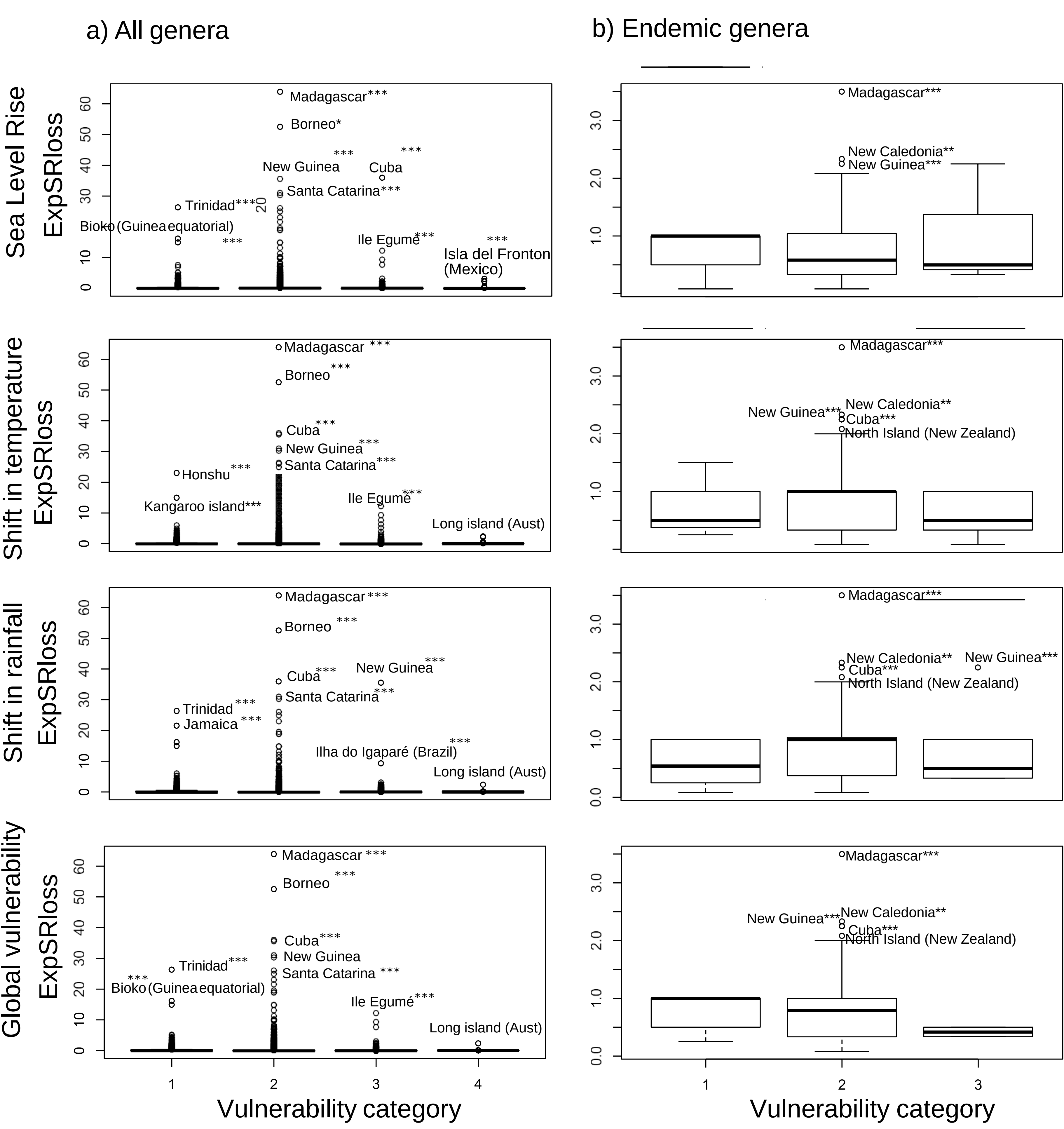

2. Expected loss of species richness in the most evolutionary distinct species

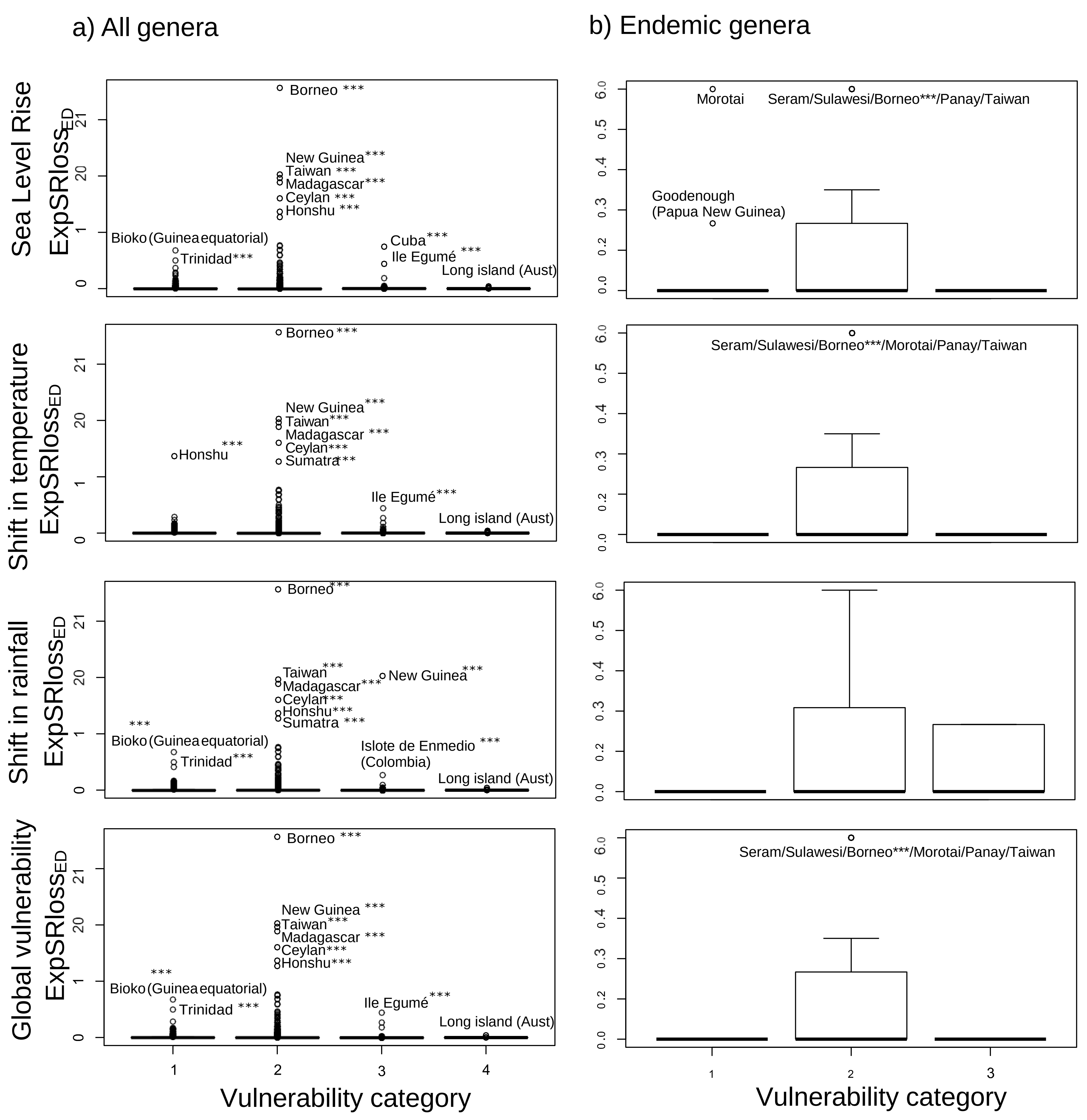

Figure S4

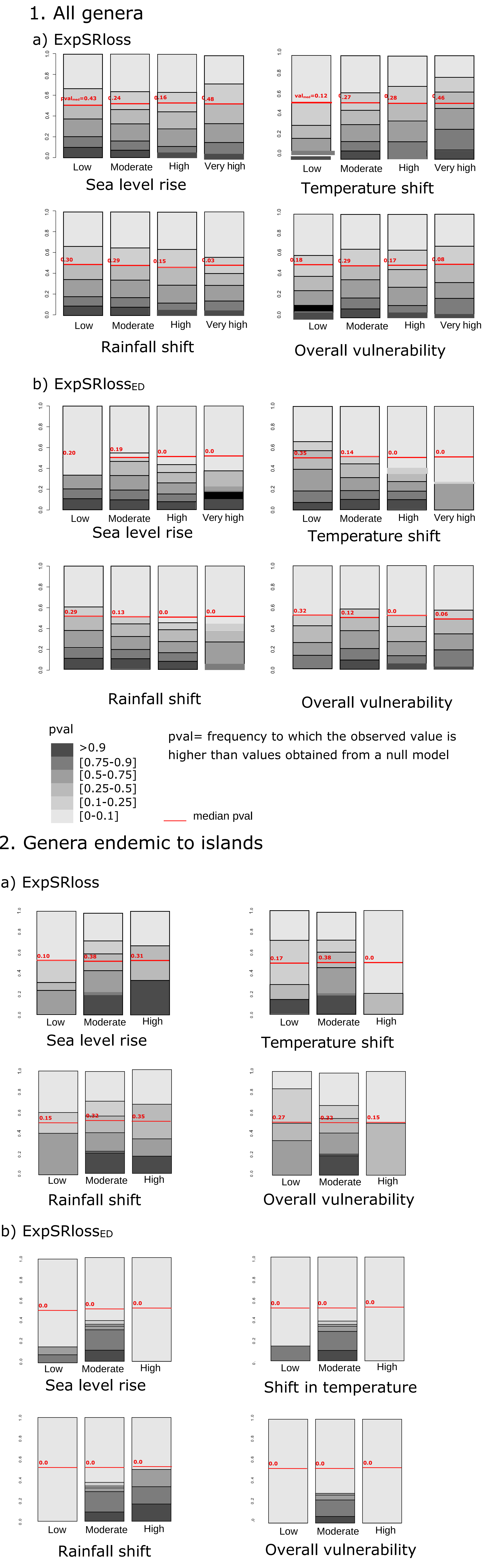
1. All genera
a) ExpSRloss
Low
Moderate
High
Very high
Sea level rise
Temperature shift
Rainfall shift
Overall vulnerability
b) ExpSRlossED
Sea level rise
Temperature shift
Rainfall shift
Overall vulnerability
pval
>0.9
[0.75-0.9]
[0.5-0.75]
[0.25-0.5]
[0.1-0.25]
[0-0.1]
pval= frequency to which the observed value is higher than values obtained from a null model
median pval
2. Genera endemic to islands
a) ExpSRloss
Sea level rise
Temperature shift
Rainfall shift
Overall vulnerability
b) ExpSRlossED
Sea level rise
Shift in temperature
Rainfall shift
Overall vulnerability

Figure S5

a) P-value$_{all}$ for ExpSRloss

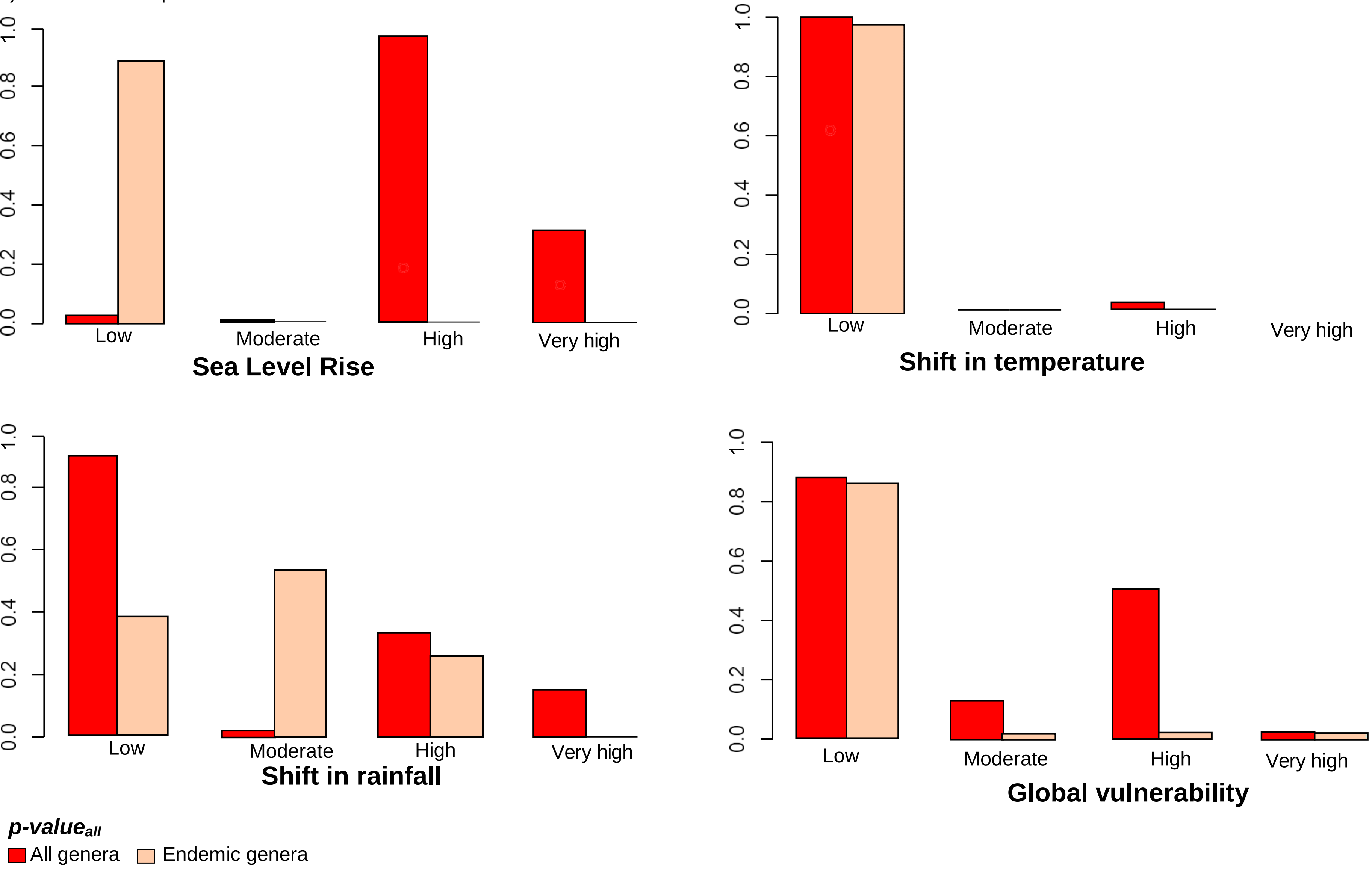

b) P-value$_{all}$ for ExpSRloss$_{ED}$

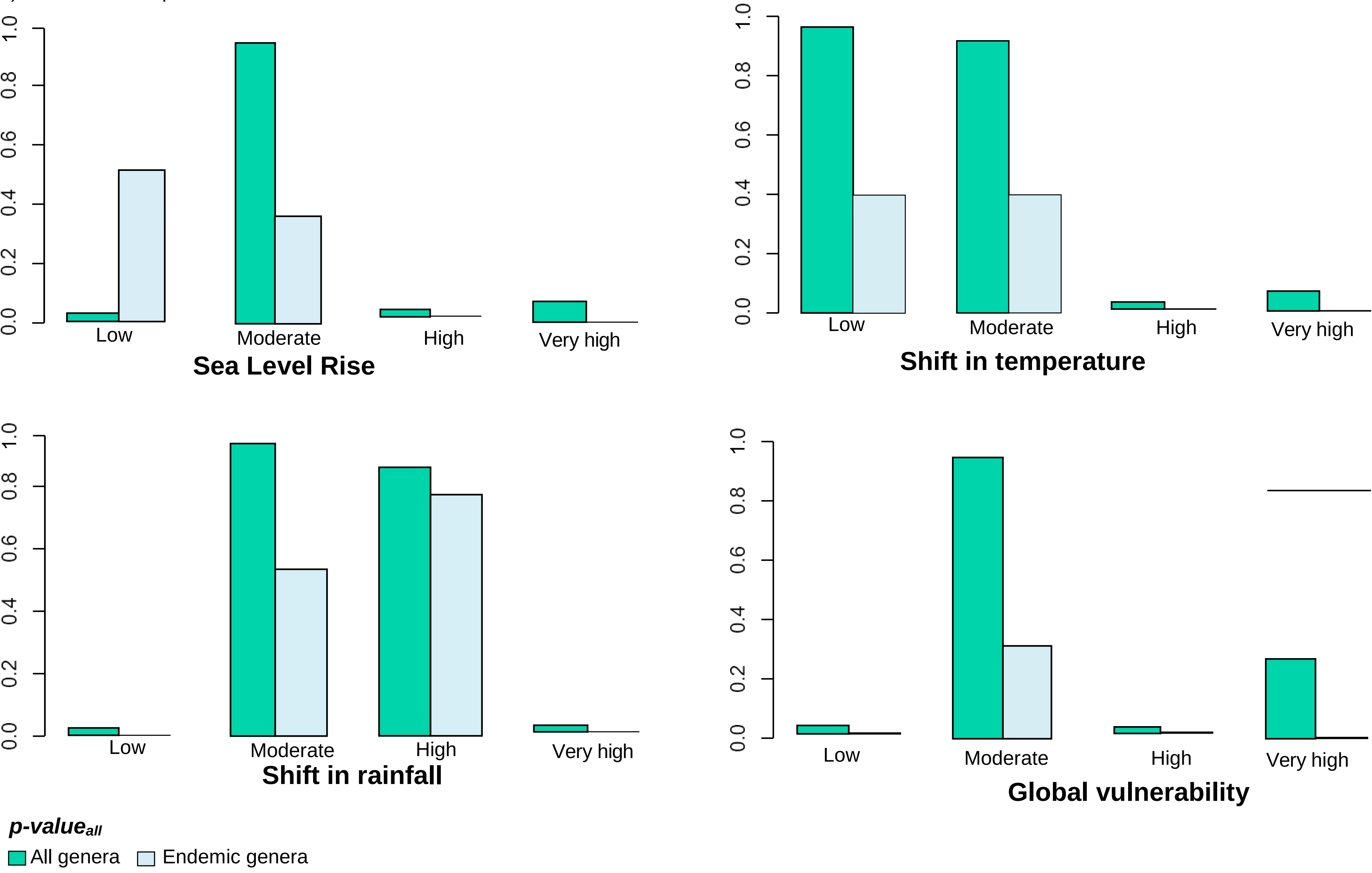

Supplement: Supplementary file 1 — Supplementary information [file 41598_2019_51107_MOESM1_ESM.pdf]
